# Implementation and evaluation of a care bundle for prevention of non-ventilator-associated hospital-acquired pneumonia (nvHAP) – a mixed-methods study protocol for a hybrid type 2 effectiveness-implementation trial

**DOI:** 10.1186/s12879-020-05271-5

**Published:** 2020-08-17

**Authors:** Aline Wolfensberger, Lauren Clack, von Felten Stefanie, Katharina Kusejko, Mirjam Faes Hesse, Werner Jakob, Dirk Saleschus, Marie-Theres Meier, Roger Kouyos, Leonhard Held, Hugo Sax

**Affiliations:** 1 Division of Infectious Diseases and Hospital Epidemiology, University Hospital Zurich, University of Zurich, Rämistrasse 100, CH-8091 Zurich, Switzerland; 2 Department of Biostatistics, Institute of Epidemiology, Biostatistics and Prevention, University of Zurich, Zurich, Switzerland; 3 Department of Medical Data Management Systems, Medical Directorate, University Hospital Zurich, Zurich, Switzerland

**Keywords:** Hospital-acquired pneumonia, Aspiration pneumonia, Infection prevention, Care bundle, Mixed-methods study, Implementation science, Qualitative research

## Abstract

**Background:**

Hospital acquired pneumonia (HAP) is divided in two distinct groups, ventilator-associated pneumonia (VAP) and non-ventilator-associated HAP (nvHAP). Although nvHAP occurs more frequently than VAP and results in similar mortality and costs, prevention guidelines and prevention focus almost exclusively on VAP. Scientific evidence about nvHAP prevention and its implementation is scarce. Therefore, we designed a mixed-methods hybrid type 2 effectiveness-implementation study to investigate both the effectiveness and implementation of a newly developed nvHAP prevention bundle.

**Methods:**

This single-centre project at the 950-bed University Hospital Zurich (UHZ) will engage the wards of nine departments with substantial nvHAP rates. The nvHAP bundle consists of five primary prevention measures: 1) oral care, 2) prevention of dysphagia-related aspiration, 3) mobilization, 4) stopping unnecessary proton pump inhibitors, and, 5) respiratory therapy. Implementation includes the engagement of department-level implementation teams, who sustain the ‘core’ intervention components of education, training, and environmental restructuring and tailor the implementation strategy to local needs. Both effectiveness and implementation outcomes will be assessed using mixed-methods. As a primary outcome, nvHAP incidence rates will be analysed by Poisson regression models to compare incidence rates before, during, and after the implementation phases (on the hospital and department level). Additionally, the association between process indicators and nvHAP incidence rates will be analysed using longitudinal Poisson regression models. A longitudinal, qualitative study and formative evaluation based on interviews, focus groups, and observations identifies supporting or hindering factors for implementation success in participating departments dynamically over time. This accumulating implementation experience will be constantly fed back to the implementation teams and thus, represents an active implementation element.

**Discussion:**

This comprehensive hybrid mixed-methods study is designed to both, measure the effectiveness of a new nvHAP prevention bundle and multifaceted implementation strategy, while also providing insights into how and why it worked or failed. The results of this study may contribute substantially to advancing knowledge and patient safety in the area of a rediscovered healthcare-associated infection - nvHAP.

**Trial registration:**

ClinicalTrials.gov: NCT03361085. Registered December 2017.

## Background

Hospital acquired pneumonia (HAP) is defined as pneumonia with first symptoms ≥48 h after admission. It is divided into two distinct groups, ventilator-associated pneumonia (VAP) and non-ventilator-associated hospital acquired pneumonia (nvHAP). Together, HAP and lower respiratory tract infections were shown to be the most common healthcare-associated infections (HAI) in both the European point prevalence study of 2011/2012 and the multistate U.S. point prevalence study in 2011 [1, 2]. In these studies, more than half of HAP - 67 and 61% - were nvHAP [1, 2]. Further, nvHAP leads to substantial morbidity and was shown to have comparable mortality and similar costs as VAP [3]. However, current research and prevention efforts still focus almost exclusively on VAP.

Scientific evidence about prevention of nvHAP is scarce and of limited quality [4]. There are no formal recommendations or evidence-based guidelines for nvHAP, and the existing HAP prevention guidelines focus almost exclusively on VAP [5–7]. In a narrative review, Passaro et al. highlighted that oral care is the most studied measure and was commonly associated with a decreased HAP rate, although a broad range of interventions are proposed [4]. Evidence is lacking for other measures such as dysphagia programs, early mobilization, and head of bed elevation [4]. The estimated proportion of preventable HAI in general ranges from 10 to 70% [8, 9], and the preventable proportion of VAP specifically was reported to be 52–55% [10, 11]. In a systematic literature review and meta-analysis about the proportion of HAI that could be prevented with multifaceted interventions only two of 132 included studies dealt with the prevention of nvHAP [9]. Hiramatsu et al. found that an outpatient bundle of nvHAP prevention measures, comprising three procedures of breathing exercises, two procedures of oral care, a procedure of nutritional control and smoking cessation prior to planned surgery, was effective to prevent postoperative pneumonia among patients with oesophageal cancer [12]. Kazaure et al. found that use of an incentive spirometer, oral hygiene with chlorhexidine, ambulation with good pain control and head-of-bed elevation to at least 30° and sitting up for all meals, accompanied by initial and ongoing education, progress reports, prevention measure documentation and order sets lead to a 43.6% decrease of postoperative pneumonia in non-cardiac surgical patients [13]. To our knowledge, there are no studies evaluating the effectiveness of an nvHAP prevention bundle on a broad patient population.

Implementation science is the scientific study of methods to promote uptake of evidence-based best practices into routine healthcare practice [14]. Although quality improvement studies often report on the effectiveness of studied interventions to improve both, process indicators and patient outcomes, little is usually reported about the context of the intervention and what factors played a role in the successful implementation of practice measures. Further, the implementation strategies used in such studies are often described in poor detail and lack theoretical justification, therefore hindering the development of an evidence base for their effectiveness [15–17]. A detailed understanding of not only what works, but also how and why it works, is helpful to ensure that evidence-based practices of proven effectiveness can be successfully replicated and implemented in other settings. To simultaneously evaluate our multifaceted implementation strategy while also testing the effectiveness of the clinical nvHAP prevention bundle, we undertake a type 2 hybrid effectiveness-implementation study [18, 19].

This comprehensive type 2 hybrid effectiveness-implementation study aims to assess the effectiveness and success factors of both, a new prevention bundle against nvHAP and a specifically designed department-based multifaceted implementation strategy in a medical and surgical patient population.

## Methods

### Aim and objectives

#### Aim

With this mixed-methods study, we aim to investigate the impact of the implementation of a newly designed nvHAP prevention bundle on the nvHAP incidence rate among inpatients in our tertiary care hospital. We will quantify the adherence to the individual bundle elements and qualitatively identify the factors that influence successful implementation.

#### Objectives

1. To determine the nvHAP bundle effectiveness on the nvHAP incidence rate
2. To determine adherence to the nvHAP bundle and each of the bundle elements
3. To relate adherence to nvHAP bundle elements with nvHAP incidence rate
4. To qualitatively monitor changes and identify trends in implementation outcomes throughout the study periods
5. To identify which factors in the implementation setting are associated with the actual degree of local implementation of the nvHAP bundle

### Study setting

The study is conducted at the University Hospital Zurich (UHZ), Switzerland, a 950-bed tertiary-care teaching hospital covering all medical specialties except paediatrics and orthopaedics.

#### Study population

All patients hospitalized in nine predefined medical and surgical departments and their corresponding wards will be included in this study. The nine departments were chosen based on the following criteria; 1) nvHAP rate above the 50th percentile according to UHZ nvHAP data from the year 2017; 2) high absolute number of patients with nvHAP according to UHZ nvHAP data from the year 2017; 3) organizational structure, e.g. departments sharing same nursing or medical personnel; 4) representing both medical and surgical specialties.

### Intervention

#### Clinical intervention: the nvHAP bundle

The University Hospital Zurich nvHAP bundle was designed by an interprofessional and interdisciplinary group of experts. Elements were chosen based on the evidence, although scarce, of their effectiveness and based on their anticipated feasibility and implementability. The bundle consists of five prevention measures (details see Additional file 1 nvHAP Bundle).

**1. Oral care**, i.e. mechanical oral care with or without pharmacological products, once daily in all patients, and three times daily in patients with swallowing difficulties.

**2. Prevention of dysphagia-related aspiration**, i.e. applying a ‘modified swallowing assessment’ (MSA) adapted from the ‘Standardized Swallowing Assessment’ by Perry et al. [20] (Additional file 2 ‘MSA Perry’) in a defined risk population, followed by further evaluation and treatment of dysphagia residing with the responsible physicians.

**3. Mobilization**, i.e. mobilization at least once at the day of surgery and at least twice daily in all other patients without contraindications.

**4. Stopping unnecessary PPI and antacids**, according to a list of indications in in-hospital guidelines.

**5. Respiratory therapy**, i.e. referral to respiratory therapists advised for a defined patient population, with a final decision at the discretion of the responsible physician.

All patients will be assessed regarding whether an active intervention of healthcare providers is indicated for each of the prevention measures at the following time points: after admission, after clinical deterioration, and after major surgery during. If yes, the prevention measure is executed according to the above description. The execution of the bundle element will be documented in the electronic medical record (EMR).

#### Implementation strategy and formative evaluation

Our multifaceted implementation strategy is designed to increase ownership and local adoption in each department by engaging local implementation teams, who establish department-specific actions tailored to local needs. This strategy is also intended to facilitate adaptability, i.e. the degree to which the intervention can be adapted to meet local needs [21, 22]. Based on an initial behavioural analysis informed by sensitizing frameworks (see below, “Implementation Frameworks”) [21, 23, 24], we identified the following as promising intervention functions to increase adherence to the nvHAP bundle: increasing knowledge and understanding about the nvHAP bundle elements through education; imparting skills through technical training; and changing the physical context to increase awareness and support performance of nvHAP measures through environmental restructuring. Whereas these intervention functions to increase adherence to the nvHAP bundle elements make up the foreseeable core intervention components, each department is encouraged to adapt the delivery of these components and to employ additional promotional components according to local context, making up the ‘adaptable periphery’ of the intervention [21].

Local implementation teams, composed of one nurse, one physician and one physiotherapist, will be established in each department. During recurrent “action plan” meetings, the local implementation team from each department, with support from the nvHAP study team, will be responsible for assessing the current implementation status with respect to each bundle element and establishing an “action plan” with a list of planned actions aimed to increase adherence to bundle elements according to local needs. Local implementation teams will be responsible for implementing the nvHAP bundle in their respective departments. Established “action plans” will be revisited to assess progress and refine necessary actions, as described below.

The nvHAP study team, based in the infection prevention department, will form a central coordinating team to provide local teams with support, example training materials, and feedback on process and outcome data. Additionally, we will employ a formative approach, during which we aim to continuously identify influences on implementation efforts (e.g. barriers and facilitators) and feed these insights back to local implementation teams to optimize the potential for implementation success [25]. This formative evaluation will occur in stages throughout the project, as described by Stetler and colleagues [25] and presented in Table 1. The formative evaluation will rely primarily on “action plan” meetings as an opportunity to feed information back to local implementation teams regarding identified barriers and facilitators to implementation and to refine implementation action plans accordingly.

**Table 1:** Formative Evaluation Stages

Stage	Aims	Concretization in current study
1. **Developmental evaluation, “diagnostic analysis”**	Assess levels of current practices and their determinants Prospectively identify potential barriers and facilitators to implementation	During the baseline period “Action Plan” interview with local implementation teams, the current state of practice for each nvHAP bundle measure will be assessed and determinants of current behaviour discussed. An “Action Plan” of promotional activities will be established, taking into account potential barriers and facilitators.
2. **Implementation-focused evaluation**	Assess discrepancies between established implementation plan and its operationalization Continually identify barriers and facilitators to implementation Refine implementation plan	During the “Action Plan” interview with local implementation teams following the implementation period, the previously established “Action Plan” will be revisited and actual vs. planned interventions assessed. Refinements to the action plan will be made as needed taking into account newly identified barriers and facilitators.
3. **Progress-focused evaluation**	Monitor and inform stakeholders about progress towards goals	During the Intervention period, feedback about nvHAP outcomes and process indicators will be fed back to local implementation teams.
4. **Interpretive evaluation**	Triangulate qualitative and quantitative data to enhance understanding of implementation results	Upon project completion, qualitative findings will be used to illuminate quantitative results and inform guidance about how the nvHAP bundle can best be implemented in further settings.

### Study design

**Fig. 1:**
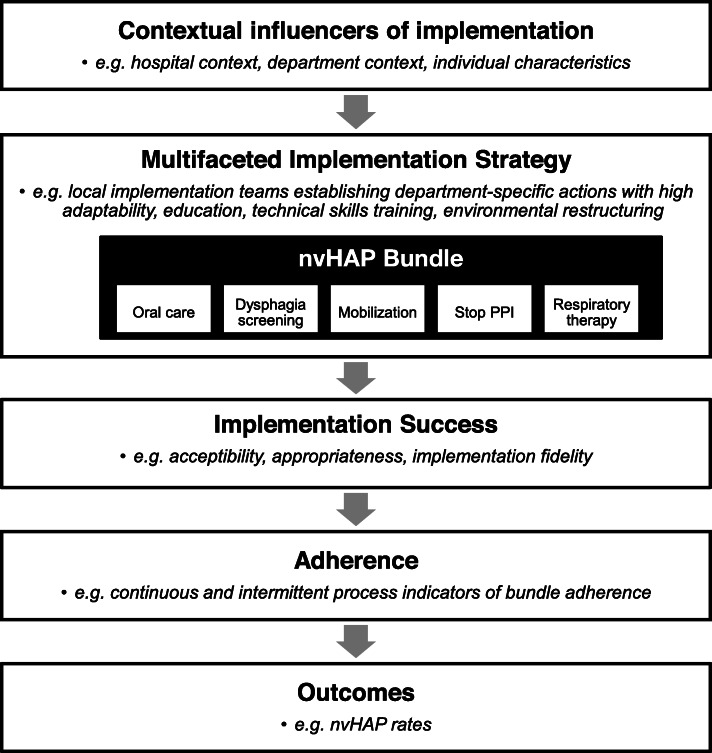
Conceptual model Legend: nvHAP = non-ventilator-associated healthcare-acquired pneumonia. This figure portrays the conceptual model of the nvHAP implementation process, in which the entire implementation process is moderated by the context in which the process is set. The contextual influencers of implementation include the larger organizational setting (i.e. the hospital and wider national context), the inner setting (i.e. the departments in which the bundle is being implemented), as well as the characteristics of individuals directly and indirectly involved in the implementation process. The contextual influencers moderate the effectiveness of specific intervention components used to implement the nvHAP bundle elements in participating departments, resulting in varying levels of implementation success, as reflected by levels of adherence to bundle components, and ultimately by the resulting outcome measures

This mixed methods study collects and analyses quantitative and qualitative data collected during the three study periods (baseline, implementation, and intervention period). The conceptual model of the study is depicted in Fig. 1.

#### Outcomes

##### Effectiveness outcomes

The primary outcome is nvHAP incidence rate, defined as the number of patients suffering from nvHAP per 1000 patient days per month. Secondary outcomes are in-hospital mortality rate; length of stay; and adherence to individual bundle elements and the nvHAP bundle as a whole.

##### Implementation outcomes

We will use a qualitative definition of implementation success composed of the following four implementation outcomes [26]: 1) acceptability, how satisfied are study participants with the intervention; 2) appropriateness, what is the perceived fit of the intervention and to what extent did participants succeed in adapting the intervention to meet the needs of their local context; 3) implementation fidelity, how closely did participants succeed in implementing the core bundle components as described in the study protocol; and 4) sustainability, to what extent did the intervention become institutionalised and anchored within ongoing operations. Implementation outcomes will primarily be assessed qualitatively through semi-structured interviews at multiple time points throughout the project, both, to assess implementation progress and to inform our formative evaluation (Table 1). Implementation fidelity will further be assessed through observation and artefact analysis by comparing planned and actual implementation activities. Quantitative data on adherence to the five bundle measures, as described below, will also be considered in assessing implementation fidelity. Sustainability will be particularly assessed by identifying examples of how the intervention has been integrated into local processes and structures such that it is likely to continue as a part of stable operations [26]. In assessing implementation outcomes at multiple time points, we aim to identify what has been described by Proctor and colleagues as “leading” and “lagging” indicators of implementation success [26] – where leading indicators are those that reflect the outcome of a change in practice early on or even predict it, and lagging indicators reflect the delay between a change in practice and the observable outcomes.

#### Study periods on department and hospital level

Baseline period will start at the same time for all departments and will be of different length (minimum 12 months) as implementation of nvHAP prevention measures will occur at the department level and the start of implementation activities is chosen by every department, primarily relying on availability of resources.

We define three study periods on the *department level*, 1) department baseline period, before implementation of nvHAP bundle in the specific department; 2) department implementation period, a two month time frame starting with the beginning of implementation activities in the respective department; 3) department intervention period following the department implementation period.

**Fig. 2:**
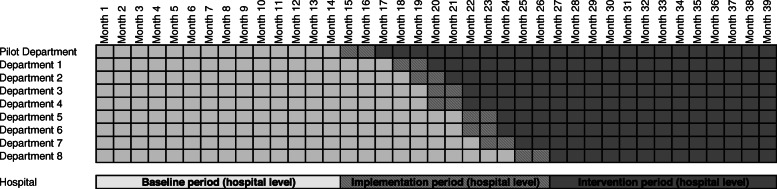
Study time table for department and hospital level (example of anticipated inclusion time points) Legend: Department and hospital baseline period (i.e. before implementation, light grey), followed by implementation period (i.e. during implementation, shaded), and intervention period (i.e. after implementation, dark grey)

On the *hospital level* the three periods are defined as follows: 1) hospital baseline period, before starting implementation in the first department; 2) hospital implementation period, from the beginning of the implementation period of the first department until end of implementation period of the last included department; 3) hospital intervention period following the hospital implementation period. Figure 2 depicts an anticipated study timetable.

**Fig. 3:**
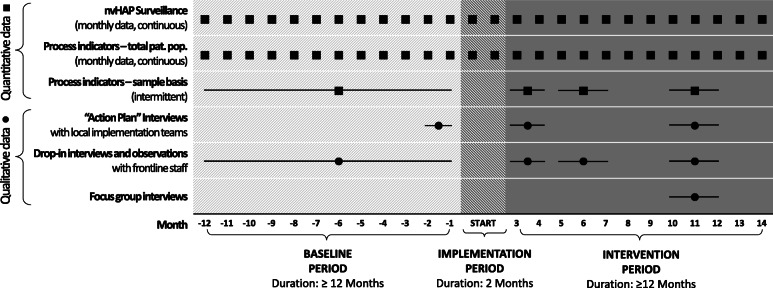
Study periods and data collection in a single exemplary department Legend: The baseline period of 12 months or longer is followed by an implementation period defined to be 2 months long, and an intervention period of again 12 months or longer. The figure depicts the data collection time points, with squares indicating quantitative data collection and circles indicating qualitative data collection time points

Quantitative and qualitative data collection will continue throughout the project and follows the study periods on the department level (Fig. 3).

The first department (pilot department) is used to test quantitative and qualitative data collection tools and the feasibility of the implementation strategy. Insights from this pilot department will help to improve the implementation strategy and study tools prior to the inclusion of further departments.

#### Implementation frameworks

Our study is theoretically informed by the Consolidated Framework for Implementation Research (CFIR) [21] and the Theoretical Domains Framework (TDF) [23]. Both the CFIR and the TDF integrate findings from theoretical literature into synthesized frameworks consisting of constructs that may mediate behaviour change [21, 23]. Whereas the TDF domains represent a set of constructs related to individual behaviour change, the CFIR domains include constructs relating to broader organizational behaviour change. For the current inquiry, we find the use of both frameworks useful to capture influencers of behaviour at the individual level, as well as the department, the overall hospital, and the wider environmental context. The CFIR and the TDF will inform the intervention implementation strategy, as previously described, and guide the qualitative data collection (semi-structured interview guides) and analyses (use of TDF as deductive coding framework). In particular, use of these sensitising frameworks throughout our study will facilitate the timely identification of barriers and facilitators and will also provide insights as to which additional intervention components are most likely to be successful in addressing the identified barriers [24].

### Data collection

#### Data sources

In the study hospital, all patient data are charted electronically via an EMR system. Selected data are stored in a clinical data warehouse.

#### Quantitative data collection

##### NvHAP surveillance

We apply the European Centre for Disease Prevention and Control (ECDC) definition criteria for pneumonia that are used in the ECDC point prevalence studies [27] (Additional file 3 ‘ECDC nvHAP definition’). In brief, the pneumonia definition comprises radiologic criteria, systemic symptoms (fever > 38 °, leukopenia or leukocytosis) and pulmonary symptoms (e.g. cough, sputum production). Pneumonia is defined as hospital-acquired, if symptoms start ≥48 h after admission. If an invasive respiratory device was present in the 48 h preceding symptom onset, the pneumonia is considered a ventilator-associated pneumonia and thus not subject of this study. A validated semi-automated surveillance system for nvHAP is used [28]. Place of nvHAP acquisition is defined as department, ward and room to which the patient was affiliated 48 h before first symptoms of nvHAP, unless shorter incubation period is evident from patient history.

##### Process indicators

Process indicators portraying adherence to the nvHAP bundle elements will be monitored in two ways. First, for all five prevention measures, at least one surrogate parameter for adherence is continuously extracted from the EMR of the *total patient population* (continuous process indicators; see Additional file 4 ‘Process indicators’). This parameter, e.g. tooth brushing provided by nurses, will be expressed per department, and month, and per hospital days. Second, we will monitor process indicators on a *sample basis* with individual assessment of a subset of 50 patients (denominator) at four different time points per department (intermittent process indicators; see Additional file 4 ‘Process indicators’). The latter allows a more detailed description of adherence, including non-documented prevention measures (e.g. oral care executed by patient) and takes into consideration the individual need of patients for the specific prevention measure (e.g. respiratory therapy is indicated only in a subset of patients). From the intermittent process indicators the ‘nvHAP adherence score’ will assess patient based adherence per department and time point. The score is based on samples of 50 patients, the ‘nvHAP adherence indicator’ takes the value 1 in the case the specific prevention measure was completed in the specific patient, 0 if that was not the case, and “empty” in the case of missing values. The ‘nvHAP adherence score’ is calculated by summing up the five proportions of patients with completed specific prevention measures (i.e. ‘nvHAP adherence indicator’=1) dividing it by factor five (Additional file 5 ‘nvHAP adherence score’).

#### Qualitative data collection

Longitudinal qualitative data will be collected throughout the project as portrayed in Fig. 3, including action plan interviews with local implementation teams, drop-in interviews with frontline staff, and focus group interviews, as described in Table 2. The researchers involved in qualitative data collection and analysis, who are also part of the implementation team, will seek to demonstrate *empathic neutrality* [29], for example by prefacing interviews with the fact that we are interested in learning about implementation experiences and that there are no right or wrong answers. In doing so, we hope to limit desirability bias in the information shared. Having three data collection activities will also allow for rigorous triangulation of findings among data sources and will all inform the ongoing formative evaluation (see Table 1).

**Table 2:** Qualitative data collection methods

Data collection method	Participants	Description	Documentation
Action plan interviews	Local nvHAP ambassadors	Semi-structured interviews of approximately one hour to assess the current implementation status of each nvHAP bundle element throughout the study periods, as well as identify potential or actual barriers and facilitators to implementation, and plan a list of actions to be taken locally.	Interviews will be audio-recorded and transcribed where acceptable and structured notes will be taken systematically. These and the written action plan documents established after each interview will be included in qualitative analysis.
Drop-in interviews	Frontline clinicians	Short, semi-structured, drop-in interviews of 10–15 min to learn from frontline staff about their experience with the nvHAP implementation and identify local barriers and enablers to implementation.	Detailed, structured notes will be taken during and after each drop-in interview and/or the interview will be audio-recorded and transcribed verbatim to be included in qualitative analysis.
Focus group interview	Frontline clinicians	Semi-structured focus group interviews of approximately 1 h to assess implementation outcomes among frontline staff.	Focus groups will be audio-recorded and transcribed verbatim for inclusion in qualitative analysis.

For drop-in and focus groups interviews, participants will be purposefully sampled to include a representative mix of professions (nurses, physicians, and physiotherapists) from wards within the participating departments Given inconsistencies in definition and application of ‘saturation’ as a measure of sufficient sampling, ‘information power’ has been proposed as a concept to guide adequate sample size [30]. By having a clearly defined qualitative study aim, an information-rich sample of interview participants, guiding theoretical frameworks to inform structured data collection by skilled interviewers expert in the study topic, our study design and sampling strategy is designed to achieve high information power [30].

### Analysis

#### Quantitative analysis

##### Analyses of nvHAP bundle effectiveness

To evaluate the effectiveness of the intervention bundle, two distinct analyses are performed. First, a change point model will be combined with piecewise constant rates with additional sine-cosine waves to account for seasonality. Poisson regression (with log link function) is used to analyse the monthly overall nvHAP incidence rate over all departments, using the monthly sum of the nvHAP cases over all departments as “count” and the monthly sum of the number of patient days (in thousands) over all departments as offset. Study period on the hospital level (hospital baseline, implementation, intervention period) will be used as explanatory factor (see Additional file 6 ‘Statistical analysis’ for detailed statistical model). We may use a quasi-poisson model in case of overdispersion.

Second, a longitudinal Poisson regression will be used. The monthly number of nvHAP cases in each department will be modelled by a generalized estimating equation (GEE) with departments as clusters. This allows to account for the non-independence of consecutive nvHAP counts within departments, to model the temporal correlation structure (e.g. first order autoregressive) and to account for over-dispersion, if necessary. We will assume a Poisson error distribution for the nvHAP counts and use the log link function. As above, we may use a quasi-Poisson model in case of overdispersion. We will use a time-dependent, department-specific binary indicator variable for department-level implementation of the intervention bundle (possibly with an intermediate level for the implementation phase) as explanatory variable. Further, we will adjust for seasonality of nvHAP incidences by inclusion of sine/cosine waves.

Because the baseline period includes nvHAP rates from 2017 which served (inter alia) as basis for the choice of the nine departments, we will perform sensitivity analyses excluding data from 2017 for all analyses described above to assess a potential “regression to the mean” effect.

##### Analyses of process indicators

To portray adherence to the single prevention measures and the nvHAP bundle as a whole a descriptive analysis will be performed, summarizing continuous and intermittent process indicators and the ‘nvHAP overall adherence score’ by department-level periods. Further, we will evaluate whether the process indicators are associated with the nvHAP incidence rate. We will use GEEs with Poisson error and departments as clusters (as described above) to model the monthly nvHAP rates as dependent on either single continuous process indicators or on all continuous process indicators combined.

To model monthly nvHAP as dependent on intermittent process indicators (either single process indicators, all process indicators combined, or the nvHAP overall adherence score), we will use GEEs with Poisson error and departments as clusters (as described above). Because the intermittently collected process indicators are collected only at four time points, we will use linear interpolation to derive monthly values for these process indicators.

#### Qualitative analysis

Longitudinal qualitative data from drop-in, action-plan and focus group interviews will be included in a cross-case analysis, where each participating department represents a case. In a first step, all interview transcripts and notes will be coded deductively using the Theoretical Domains Framework (TDF) as a coding scheme as well as additional codes to capture our pre-defined implementation outcomes [23]. Inductive thematic analysis will then be conducted to identify themes relevant to the implementation within TDF domains. Analyses will begin with at the case level to understand the local influencers of implementation at the department level, allowing us, for example, to assess how implementation outcomes shift over time in relation to the undertaken interventions and in light of local barriers and facilitators. Then, cross-case matrices will be used to explore any trends across departments [31]. This qualitative analysis will allow us to make a qualitative assessment about which local factors and interventions contributed to implementation success. Our in-depth findings will also help to ultimately shine light on quantitative study results. The researchers involved in qualitative analysis will engage in an ongoing process of *reflexivity* [29], considering the role of our own preconceptions and close relation to the implementation process, while also aiming to provide an authentic account of the implementation process.

## Discussion

With this mixed-methods study we will close critical knowledge gaps about the prevention of nvHAP, a neglected but common HAI. To date, literature about prevention measures against nvHAP is scarce [4], and our study will provide further knowledge by assessing the effectiveness of a five element prevention bundle against nvHAP on lowering nvHAP incidence rates. To our knowledge, it is the first study testing an inpatient bundle of nvHAP prevention measures on a broad patient population. Moreover, as effective implementation is as important as choosing the right bundle elements [14, 32], we place focus on a theoretically-informed implementation strategy.

The quantitative part of the study aims to not only measure the primary outcome parameter nvHAP incidence rate over time, but to also measure process indicators. This will help us to better understand if the implementation process was successful and to evaluate direct association between prevention measures and nvHAP incidence rate. As the nvHAP bundle cannot be effective if it is not well implemented, it is important to also measure implementation outcomes (e.g. acceptability, appropriateness, fidelity, and sustainability) as necessary preconditions for achieving the desired changes in clinical outcomes.

A major strength of this study is the mixed-methods approach, including an extensive formative qualitative study to provide insights about how and why departments succeeded, or faced challenges, in implementing the nvHAP bundle. With some notable exceptions [33–36], many qualitative implementation evaluations are limited to inquiries conducted at a single point in time. Such inquiries are prone to participant recall biases and may be insufficient to telling the whole implementation story [26]. Our longitudinal qualitative study aims to provide critical contextual insights to guide others hoping to implement the nvHAP bundle. Additionally, the participatory approach of our formative evaluation is intended to increase project commitment among stakeholders, particularly local implementation teams.

The limitations of our study are the following: First, our study does not include a control group. We abstained from conducting a randomized controlled trial due to anticipated high contamination between departments/wards within the same hospital. Second, the duration of the implementation period is determined to be 2 months not accounting for possibly longer duration due to the formative approach of the implementation strategy. We aim to address this point by analysing the results both on the hospital and department level. Third, by continuously collecting process indicators from EMR, we cannot preclude reporting bias (e.g. increased documentation of oral care). We address this issue by additionally measuring process indicators on an individual basis. Further, although we take efforts to demonstrate empathic neutrality during our qualitative data collection, we cannot entirely preclude the possibility that qualitative researchers may be perceived as being partial, leading to potential desirability bias in the qualitative data. Finally, we acknowledge that our formative process evaluation does in itself lead to changes in implementation plans and that these changes must be documented with great care to keep track of the exact implementation activities. Rather than purely a limitation, we view this as a strength of our study, and we anticipate that it should also be integrated into recommendations for those wishing to replicate results of our future nvHAP study.

In conclusion, with this innovative mixed-methods study design, we will assess the effectiveness of the nvHAP bundle, but also measure process indicators of the nvHAP bundle and contextual factors influencing implementation uptake. We will be able to triangulate our findings, i.e. correlate nvHAP rates with adherence data of the prevention bundle and again with qualitative measures of implementation success. Further, our mixed-method approach will be of great value to understanding the complex contextual interactions that influence implementation success, which are necessary to inform implementation guidance for other institutions planning to implement the nvHAP bundle.

Addendum: Due to the COVID-19 pandemic, the study data collection had to be terminated earlier than planned (i.e. end of February 2020). Additional file 7 informs about the details of early study termination.

## Supplementary information

**Additional file 1.** nvHAP Bundle.

**Additional file 2.** Modified swallowing assessment (MSA).

**Additional file 3.** ECDC Definition for nvHAP.

**Additional file 4.** Process indicators.

**Additional file 5.** nvHAP adherence score.

**Additional file 6.** Statistical Analysis.

**Additional file 7.** Addendum: Early study termination.

## Data Availability

Data sharing is not applicable to this article as no datasets were generated or analyzed during the current study.

## Abbreviations

CFIR: Consolidated Framework for Implementation Research
ECDC: European Centre for Disease Prevention and Control
EMR: Electronic medical record
GEE: Generalized estimating equation
HAI: Healthcare-associated infections
HAP: Hospital-acquired pneumonia
ICU: Intensive care unit
MSA: Modified swallowing assessment
nvHAP: Non-ventilator-associated hospital-acquired pneumonia
PPI: Proton pump inhibitors
TDF: Theoretical Domains Framework
UHZ: University Hospital Zurich
VAP: Ventilator-associated pneumonia
