# Correction to: Implementation and evaluation of a care bundle for prevention of non-ventilator associated hospital-acquired pneumonia (nvHAP) – a mixed-methods study protocol for a hybrid type 2 effectiveness-implementation trial

**DOI:** 10.1186/s12879-021-05800-w

**Published:** 2021-01-25

**Authors:** Aline Wolfensberger, Lauren Clack, Stefanie von Felten, Katharina Kusejko, Mirjam Faes Hesse, Werner Jakob, Dirk Saleschus, Marie-Theres Meier, Roger Kouyos, Leonhard Held, Hugo Sax

**Affiliations:** 1 Division of Infectious Diseases and Hospital Epidemiology, University Hospital Zurich, University of Zurich, Rämistrasse 100, CH-8091 Zurich, Switzerland; 2 Department of Biostatistics, Institute of Epidemiology, Biostatistics and Prevention, University of Zurich, Zurich, Switzerland; 3 Department of Medical Data Management Systems, Medical Directorate, University Hospital Zurich, Zurich, Switzerland

**Correction to: BMC Infect Dis (2020) 20:603**

**https://doi.org/10.1186/s12879-020-05271-5**

After publication of the original article [1], the authors identified an error in two authors’ names of Stefanie von Felten and , Mirjam Faes Hesse. The given name and family name were erroneously transposed.

The incorrect author name is:

Stefanie von Felten ⋄ given name: von Felten; family name: Stefanie

Mirjam Faes Hesse ⋄ given name: Mirjam Faes; family name: Hesse

The correct author name is:

Stefanie von Felten ⋄ given name: Stefanie; family name: von Felten

Mirjam Faes Hesse ⋄ given name: Mirjam; family name: Faes Hesse

The original article has been corrected.

## References

[1] Wolfensberger A (2020). Implementation and evaluation of a care bundle for prevention of non-ventilator associated hospital-acquired pneumonia (nvHAP) – a mixed-methods study protocol for a hybrid type 2 effectiveness implementation trial. BMC Infect Dis.

