# Supplementary material for: Implementation and evaluation of a care bundle for prevention of non-ventilator-associated hospital-acquired pneumonia (nvHAP) – a mixed-methods study protocol for a hybrid type 2 effectiveness-implementation trial
Source: BMC Infect Dis. 2020 Aug 17;20:603. doi: 10.1186/s12879-020-05271-5 (PMC7429945; doi:10.1186/s12879-020-05271-5)
Supplement: Supplementary file 1 — Additional file 1. nvHAP Bundle. [file 12879_2020_5271_MOESM1_ESM.docx]

**Annex nvHAP Bundle**

**1. Oral care**. Mechanical oral care (e.g. tooth brushing) at least once a day, either executed by the patient him-/herself if a good quality of oral care is guaranteed, or otherwise executed or assisted by a nurse. Patients with dysphagia warrant mechanical oral care three times a day. Pharmacological oral care with chlorhexidine is indicated for patients with defined pathologies of the mouth, e.g. severe gingivitis or periodontitis. Referral to dental treatment is prescribed by the treating physician if indicated.

**2. Prevention of dysphagia-related aspiration**. A ‘modified swallowing assessment’ (MSA) adapted from the ‘Standardized Swallowing Assessment’ by Perry et al. is used for dysphagia bedside screening (see Annex MSA). Screening with MSA will be executed on every patient fulfilling at least one of the following criteria: 1) neurological or neuromuscular disease; 2) after major thoracic, abdominal, or facio-oral surgery; 3) need for assisted oral care. If MSA screening indicates ‘risk for aspiration’ the patient is referred to a facio-oral tract therapist or a speech therapist for further evaluation and treatment. Until further evaluation, the patient should ideally have ‘nil per os’ (no oral intake) or, as a minimum requirement, a structure modified diet. Treatment or further evaluation of dysphagia resides with the responsible physicians, nurses, and therapists and can comprise videofluoroscopy or functional endoscopic evaluation to further evaluate the swallowing problem, and swallowing exercises, oral stimulation, structure modified diet, or enteral and parenteral nutrition.

**3. Mobilization**. Every patient without contraindication requires mobilization out of bed or at the bedside at least twice daily. Early postoperative mobilization (i.e. mobilization out of bed or at the bedside at the day of surgery) is indicated after surgery. Mobilization can either be executed by the patient himself or assisted by nurses or physiotherapists.

**4. Stopping PPI and antacids if not indicated**. PPI or antacids without indication should be suspended as soon as possible.

**5. Respiratory therapy**. Responsible physicians are advised to consider and refer to respiratory therapy for patients fulfilling at least one of the following criteria: 1) patients with chronic pulmonary disease; 2) patients needing >3 litre of oxygen for oxygen saturation of >93%; 3) patients after abdominal/thoracic surgery or –injury; 4) patients not staying out of bed >4 hours per day; 5) patients having problems with coughing and are at risk for accumulating bronchial secretions. Respiratory therapy resides with the physiotherapist and can comprise e.g. manual or assisted respiratory therapy and mucolysis, therapeutic body positioning, and activation and mobilization.
