# Supplementary material for: Implementation and evaluation of a care bundle for prevention of non-ventilator-associated hospital-acquired pneumonia (nvHAP) – a mixed-methods study protocol for a hybrid type 2 effectiveness-implementation trial
Source: BMC Infect Dis. 2020 Aug 17;20:603. doi: 10.1186/s12879-020-05271-5 (PMC7429945; doi:10.1186/s12879-020-05271-5)
Supplement: Supplementary file 2 — Additional file 2. Modified swallowing assessment (MSA). [file 12879_2020_5271_MOESM2_ESM.docx]

**Annex Modified swallowing assessment (MSA)**

We developed a bedside screening tool for dysphagia by adapting the ‘Standardized Swallowing Assessment’ by Perry et al., by only using elements of the ‘pre-swallow screening checklist’. Our ‘modified swallowing assessment’ (MSA) consists of the following six items:

1) Is the patient awake and alert, or responding to speech?

2) Can the patient cough when asked to?

3) Is the patient able to maintain some control of his saliva?

4) Is the patient able to lick top and bottom lip?

5) Is the patient able to breathe freely?

6) Is a wet or hoarse-sounding voice absent?

If the answer to one or more than one item is ‘No’, the patient is considered to be ‘at risk for aspiration’.
