# Supplementary material for: Implementation and evaluation of a care bundle for prevention of non-ventilator-associated hospital-acquired pneumonia (nvHAP) – a mixed-methods study protocol for a hybrid type 2 effectiveness-implementation trial
Source: BMC Infect Dis. 2020 Aug 17;20:603. doi: 10.1186/s12879-020-05271-5 (PMC7429945; doi:10.1186/s12879-020-05271-5)
Supplement: Supplementary file 3 — Additional file 3. ECDC Definition for nvHAP. [file 12879_2020_5271_MOESM3_ESM.docx]

**Annex ECDC Definition for nvHAP**

Two or more serial chest X-rays or CT-scans with a suggestive image of pneumonia for patients with underlying cardiac or pulmonary disease (in patients without underlying cardiac or pulmonary disease one definitive chest Xray or CT-scan is sufficient),

and at least one of the following:

- fever > 38 °C with no other cause;

- leukopenia (<4 000 WBC/mm3) or leucocytosis (≥ 12 000 WBC/mm3)

and at least one of the following (or at least two if clinical pneumonia only = PN 4 and PN 5):

- new onset of purulent sputum, or change in character of sputum (colour, odour, quantity, consistency)
- cough or dyspnea or tachypnea
- suggestive auscultation (rales or bronchial breath sounds), ronchi, wheezing
- worsening gas exchange (e.g. O2 desaturation or increased oxygen requirements or increased ventilation demand)

and according to the used diagnostic method:

1. Bacteriologic diagnostic test performed by:

- Positive quantitative culture from minimally contaminated LRT (lower respiratory tract) specimen (PN 1):
  - broncho-alveolar lavage (BAL) with a threshold of > 104 CFU2/ml or ≥5 % of BAL obtained cells contain intracellular bacteria on direct microscopic exam (classified on the diagnostic category BAL);
  - protected brush (PB Wimberley) with a threshold of > 103 CFU/ml;
  - distal protected aspirate (DPA) with a threshold of > 103 CFU/ml.
- Positive quantitative culture from possibly contaminated LRT specimen (PN 2):
  - Quantitative culture of LRT specimen (e.g. endotracheal aspirate) with a threshold of 106 CFU/ml

1. Alternative microbiology methods (PN 3)

- positive blood culture not related to another source of infection;
- Positive growth in culture of pleural fluid;
- pleural or pulmonary abscess with positive needle aspiration;
- histologic pulmonary exam shows evidence of pneumonia;
- positive exams for pneumonia with virus or particular germs ( Legionella , Aspergillus , mycobacteria, mycoplasma, Pneumocystis carinii )
  - positive detection of viral antigen or antibody from respiratory secretions (e.g. EIA, FAMA, shell vial assay, PCR);
  - positive direct exam or positive culture from bronchial secretions or tissue;
  - seroconversion (e.g. influenza viruses, Legionella , Chlamydia );
  - detection of antigens in urine ( Legionella ).

1. Others:

- positive sputum culture or non-quantitative LRT specimen culture (PN 4);
- no positive microbiology (PN 5).

Notes: One definitive chest X - ray or CT - scan for the current pneumonia episode may be sufficient in patients with underlying cardiac or pulmonary disease if comparison with previous X - rays is possible. PN 1 and PN 2 criteria were validated without previous antimicrobial therapy. However, this does not exclude the diagnosis of PN 1 or PN 2 in the case of previous antimicrobial use.
