# Supplementary material for: Implementation and evaluation of a care bundle for prevention of non-ventilator-associated hospital-acquired pneumonia (nvHAP) – a mixed-methods study protocol for a hybrid type 2 effectiveness-implementation trial
Source: BMC Infect Dis. 2020 Aug 17;20:603. doi: 10.1186/s12879-020-05271-5 (PMC7429945; doi:10.1186/s12879-020-05271-5)
Supplement: Supplementary file 4 — Additional file 4. Process indicators. [file 12879_2020_5271_MOESM4_ESM.docx]

**Annex Process indicators**

1. **Continuous process indicators (collected on department level in total patient population)**

**Oral care**. Documented nurse-assisted executions of oral care per 1000 patient days.

**Prevention of dysphagia-related aspiration**. Execution of MSA dysphagia screening per 1000 patient days.

**Mobilization**. Documented nurse-assisted mobilizations per 1000 patient days and physiotherapy sessions per 1000 patient days.

**Stopping PPI and antacids if not indicated**. Consumption of ‘Drugs for Acid related disorders’ (ATC group A02) according to the WHO Anatomical Therapeutic Chemical (ATC) and its subgroups ‘Antacids’ (ATC group A02a) and ‘Drugs for peptic ulcer and gastro-oesophageal reflux disease’ (ATC group A02b) per 1000 patient days.

**Respiratory therapy**. Physiotherapy sessions per 1000 patient days and use of EzPAP® (Positive Airway Pressure System) per 1000 patient days.

1. **Intermittent process indicators (collected on a sample basis at four different time points per department)**

**Oral care**. Data collection happens for the preceding day. The EMR is consulted for documented mechanical oral care (i.e. tooth brushing) either provided by nurses or executed by the patient himself. The presence of dysphagia is assessed. If necessary, the patient is visited and questioned about oral care. Adherence to ‘oral care’ is considered accomplished if a patient not suffering from dysphagia had at least one oral care episode, or a patient suffering from dysphagia had three oral care episodes.

**Prevention of dysphagia-related aspiration**. Data collection happens after patient discharge overseeing the total hospital stay. The EMR is assessed for appointments with facio-oral tract therapist or a speech therapist, and predefined medical indications for screening for dysphagia (i.e. need for assisted oral care, neurological or neuromuscular disease, or thoracic, cardiac, and facio-oral surgery). Execution and result of MSA screening for dysphagia, and the prescribed diet until screening is recorded. Adherence to this bundle element is considered accomplished if the patient either had no indication for screening, had dysphagia screening, or had an appointment with facio-oral tract therapist or a speech therapist. In patients ‘at risk for aspiration’ the prescribed diet from dysphagia screening to consultation with facio-oral tract therapist or a speech therapist has to be either ‘nil per os’ or texture modified.

**Mobilization**. Data collection happens for the preceding day. The EMR is screened for documented mobilization either provided by nurses/physiotherapists, or by the patient himself. The presence of surgical procedure at the preceding day, contraindications for mobilization, and a daily assessed nursing score about activity level of the patient (ePA-AC, ‘ergebnisorientierte PflegeAssessment in acute care’, in English, ‘performance-orientated nursing assessment in acute care’) are recorded. If necessary, the patient is visited and questioned about mobilization during the preceding day. Adherence to ‘mobilization’ is considered accomplished if the patient had mobilization at least once at the day of surgery, twice at every other day, had a normal activity level, or had contraindications for mobilization.

**Stopping PPI and antacids if not indicated**. Data collection happens after patient discharge overseeing the total hospital stay. The EMR is consulted to assess if patient had PPI or antacids (according to the WHO Anatomical Therapeutic Chemical (ATC), ‘Drugs for Acid related disorders’, code A02) during his stay, if an indication for the medication was documented, and if the PPI/antacids were stopped during hospital stay. Adherence to ‘Stopping PPI and antacids if not indicated’ is considered accomplished if the patient either never had PPI/antacid, PPI/antacid were stopped during hospital stay, or if there was an indication for PPI/antacid.

**Respiratory therapy**. Data collection happens after patient discharge overseeing the total hospital stay. The EMR is screened for the medical indications for respiratory therapy (i.e. presence of chronic pulmonary disease, abdominal or thoracic surgery or injury, oxygen saturation <93% with oxygen supply of >3l/min) and the execution of physiotherapy. Adherence to ‘Respiratory therapy’ is considered accomplished if the patient either had physiotherapy (in the UHZ respiratory therapy is performed by physiotherapists) or did not have an indication for respiratory therapy.
