# Supplementary material for: Implementation and evaluation of a care bundle for prevention of non-ventilator-associated hospital-acquired pneumonia (nvHAP) – a mixed-methods study protocol for a hybrid type 2 effectiveness-implementation trial
Source: BMC Infect Dis. 2020 Aug 17;20:603. doi: 10.1186/s12879-020-05271-5 (PMC7429945; doi:10.1186/s12879-020-05271-5)
Supplement: Supplementary file 5 — Additional file 5. nvHAP adherence score. [file 12879_2020_5271_MOESM5_ESM.docx]

**Annex nvHAP adherence score**

Example for calculating the ‘nvHAP adherence-score’

|  | Oral care | Prevention of dysphagia-related aspiration | Mobilization | Stopping unnecessary PPI and antacids | Respiratory therapy |
| --- | --- | --- | --- | --- | --- |
| Patient 1 | 0 | 1 | - | 1 | 1 |
| Patient 2 | - | - | 1 | 1 | 1 |
| Patient 3 | 1 | 0 | 1 | 0 | - |
| … |  |  |  |  |  |
| Patient 50 | 0 | 1 | 1 | 1 | 1 |
| Proportion of patients with completed prevention measures | 32/45 | 20/50 | 48/50 | 42/50 | 24/32 |

The ‘nvHAP adherence score’ is calculated by summing up the five proportions of executed prevention measures (i.e. nvHAP adherence indicator = 1) and dividing it by factor five. In the above example it would be (32/45 + 20/50 + 48/50 + 42/50 + 24/32)/5 = 0.732.
