# Supplementary material for: Implementation and evaluation of a care bundle for prevention of non-ventilator-associated hospital-acquired pneumonia (nvHAP) – a mixed-methods study protocol for a hybrid type 2 effectiveness-implementation trial
Source: BMC Infect Dis. 2020 Aug 17;20:603. doi: 10.1186/s12879-020-05271-5 (PMC7429945; doi:10.1186/s12879-020-05271-5)
Supplement: Supplementary file 6 — Additional file 6. Statistical Analysis. [file 12879_2020_5271_MOESM6_ESM.docx]

**Annex Statistical Analysis**

The model of the poisson regression may be described as follows:

log(λ_t_) ~ b_0_ + b_1_*I_impl_ + b_2_*I_interv_ + c_1_*sin(ω*t) + c_2_*cos(ω*t) + log(offset)

y_t_~Poisson(λ_t_)

Where λ_t_ is the expected number of nvHAP cases in month t, b_0_ is the mean number of nvHAP cases in the baseline period (intercept), b_1_ is the change in the mean number of nvHAP cases between implementation period and baseline and b_2_ is the change in the mean number of nvHAP cases between intervention period and baseline. I_impl_ and I_interv_ are binary indicators for the implementation and intervention period. c_1_ and c_2_ are the coefficients for the sine and cosine waves, where ω = π/6 for monthly data. We assume that the observed number of nvHAP cases in month t, y_t_, is Poisson distributed with mean λ_t_. In case of over-dispersion we will use a quasi-Poisson model instead. The primary aim of the study is to quantify the multiplicative effect exp(b_2)_ (and possibly exp(b_1)_)) and to investigate if there is evidence that this effect is different from 1.
