# Supplementary material for: Implementation and evaluation of a care bundle for prevention of non-ventilator-associated hospital-acquired pneumonia (nvHAP) – a mixed-methods study protocol for a hybrid type 2 effectiveness-implementation trial
Source: BMC Infect Dis. 2020 Aug 17;20:603. doi: 10.1186/s12879-020-05271-5 (PMC7429945; doi:10.1186/s12879-020-05271-5)
Supplement: Supplementary file 7 — Additional file 7. Addendum: Early study termination. [file 12879_2020_5271_MOESM7_ESM.docx]

Addendum

Implementation and evaluation of a care bundle for prevention of non-ventilator-associated hospital-acquired pneumonia (nvHAP) – a mixed-methods study protocol for a hybrid type 2 effectiveness-implementation trial

In the study protocol we describe the duration of the study periods for each of the nine included departments as 12 months or longer for both the baseline period and the intervention period. Due to the COVID-19-pandemic, we had to terminate the data collection of our study earlier than planned, i.e. end of February 2020.

These were the three most important reasons for early termination:

- Change of patient mix: non-urgent admissions were stopped in march 2020, when cases of COVID-19 were on a steep rise in Switzerland. Consequently, patient mix was expected to be skewed towards sicker patients with higher nvHAP-risk.
- Reorganisation of wards: during the COVID-19-pandemic, wards were re-organised in terms of affiliation to department. Additionally, ward teams were pulled apart. As implementation of nvHAP-prevention measures happened on a department/ward-basis we expected a major impact on adherence to nvHAP-prevention measures
- New standard precautions: due to the COVID-19-pandemic new standard-precautions were implemented throughout the hospital. These measures were considered to potentially interfere with nvHAP-prevention measures, e.g. patients were not allowed to leave their bedplace without wearing a mask, with negative effects on prevention measures “mobilization” and “prevention of dysphagia-related aspiration”.

Due to early termination of data collection, the duration of the intervention period of three of nine departments is less than 12 months. The department that was last included has an intervention period of three months.
